# First Serological Study Revealing High Humoral Response and Evidence for Antigenic Heterogeneity in *Leishmania donovani* Induced CL in Sri Lanka

**DOI:** 10.1155/2020/5271657

**Published:** 2020-09-29

**Authors:** Bhagya Deepachandi, Sudath Weerasinghe, Samantha Ranasinghe, Thisira P. Andrahennadi, Mahendra N. Wickramanayake, Shantha Siri, Nadira Karunaweera, Vishvanath Chandrasekharan, Mitali Chatterjee, Preethi Soysa, Yamuna Siriwardana

**Affiliations:** ^1^Department of Parasitology, Faculty of Medicine, University of Colombo, Colombo 00800, Sri Lanka; ^2^Ministry of Healthcare and Nutrition, Colombo 01000, Sri Lanka; ^3^Department of Biochemistry and Molecular Biology, Faculty of Medicine, University of Colombo, Colombo 00800, Sri Lanka; ^4^Department of Chemistry, Faculty of Science, University of Colombo, Colombo 00300, Sri Lanka; ^5^National Science Foundation, 47/5 Maitland Place, Colombo 00700, Sri Lanka; ^6^Department of Pharmacology, Institute of Postgraduate Medical Education and Research, 244 B, JC Bose road, 700020, Kolkata, India

## Abstract

Posing a threat to the ongoing leishmaniasis elimination efforts in the Indian subcontinent, *L. donovani*-induced cutaneous leishmaniasis (CL) has been recently reported in many countries. Sri Lanka reports a large focus of human cutaneous leishmaniasis (CL) caused by *Leishmania donovani, a* usually visceralizing parasite. Enhanced case detection, early treatment, and in-depth understanding of sequalae are required to contain the spread of disease. Visceralizing potential of dermotropic strains has not been fully ruled out. Sri Lankan strains have shown a poor response to established serological assays. The present concern was to develop an in-house serological assay and to determine the seroprevalence of CL for identifying visceralizing potential and its usefulness in enhancing case detection. Crude cell lysate of dermotropic *L. donovani* promastigotes-based indirect enzyme-linked immunosorbent assay (ELISA) was previously optimized. Assay was evaluated using sera from 200 CL patients, 50 endemic and 50 nonendemic healthy controls, 50 patients with other skin diseases, and 50 patients with other systemic diseases. Seroprevalence and clinicoepidemiological associations were analyzed. Assay was compared with light microscopy (LM) and *in vitro* culturing (IVC). Cost comparison was carried out. Seroprevalence of CL was 82.0%. The assay had 99.5% specificity, and all healthy controls were negative at 0.189 cut-off. Positive and negative predictive values were 99.4% and 84.7%, respectively. Positivity obtained in ELISA was comparable to LM and higher than that of IVC. Cost per patient was 3.0 USD for both ELISA and LM and 6.0 USD for IVC. Infections occurring in all age groups and both genders demonstrated >75.0% of seropositivity. Patients had lesions with different durations/types/sizes showed >70.0% of seropositivity. Study identified a high seroprevalence of *L. donovani*-induced CL for the first time, indicating potential for visceralization or transient serological response. This can be used as a second line test in LM-negative CL cases to enhance clinical case detection. Further studies are warranted to examine in-depth correlations, antigen profiles, comparison with other established serological tools, and usefulness in the detection of asymptomatic cases. *(National patent LK/P/1/19697).*

## 1. Background

Leishmaniasis, one of the neglected vector-borne parasitic diseases is caused by different species of the genus *Leishmania*. Clinical manifestations are largely species-dependent, present three main entities, and varied from self-healing CL to potentially fatal MCL and VL [1].

Sri Lanka is a recent focus of human leishmaniasis in South Asia. This country reports a world's large epidemic of CL caused by *L. donovani*, a usually visceralizing and the most virulent species of the genus [2, 3]. It is found to be genetically different from other established *L. donovani* strains in the world [3–5]. The clinicoepidemiological pattern of local diseases presents great variation, showing micro changes within the CL profile [6], atypical CL as a different clinical entity [7], and poorly responding cases [8]. Furthermore, expanding spatial dimensions [6] and presence of different and independent disease foci within the country [9] were identified recently, calling for urgent action. Few cases of MCL and VL have also been reported so far [10–12].

Meanwhile, *L. donovani*-induced CL is increasingly reported in many other settings [13–15]. In-depth study of this clinical entity, enhanced early detection of asymptomatic and clinical cases, and evidence-based interventions are necessary to contain disease spread. Due to the immunogenic nature or aggressiveness of the involved parasite, systemic involvement in *L. donovani*-induced CL cannot be fully excluded without proper studies.

Serological assays are increasingly used in detecting asymptomatic and clinical infections with leishmaniasis [16–18]. However, previous local studies have demonstrated regional variations in epidemiology and poor response of a limited number of local leishmanial infections to standard rK39 assay, indicating the possibility of antigenic variation and the need for an in-house tool for serological assessment of local infections [19, 20]. Furthermore, the availability of such a tool is important for the detection of microscopy negative cases. Currently, IVC and polymerase chain reaction (PCR) are used with limited availability for the diagnosis of such cases in Sri Lanka [21, 22].

The current study evaluated an in-house ELISA, examined the serological response in *L. donovani*-induced CL infections, and evaluated the usefulness of the same as a diagnostic tool.

## 2. Methods

### 2.1. Serum Samples

Sera from five major groups of patients were used for this study.

*Group I*: Cases of CL (*n* = 200) which were confirmed for CL by LM and/or IVC and/or PCR [22–24]. Parasite materials of a small group of CL from selected cases (*n* = 15/200) were confirmed as *L. donovani* in previous genetic studies carried out in home laboratory [3–5], assuming all were caused by *L. donovani.*

*Group II*: Patients admitted to the Dermatology ward at the National Hospital of Sri Lanka (*n* = 50). All had a confirmed diagnosis for other skin diseases which mimic CL (non-CL/NCL) including immune disorders (contact dermatitis, photodermatitis, prominent hand dermatitis, and exfoliative dermatitis, (*n* = 36)) and infections (leprosy, fungal infections, tuberculosis, or bacterial infections (*n* = 14)).

*Group III*: Consisted of patients with other systemic features or diseases (NVL, *n* = 50). This group included pyrexia of unknown origin (*n* = 12), hepatomegaly/or splenomegaly (*n* = 7), viral infections (viral fever, dengue, *n* = 12), leptospirosis (*n* = 5), bacterial infections (TB, paratyphoid, brucellosis, *n* = 9), and *n* = 5 from infectious myonecrosis, bowel carcinoma, infective endocarditis, and systemic lupus erythematosus.

*Group IV*: Healthy persons lived in a disease-free area, Western Province (nonendemic healthy controls/NEHC, *n* = 50).

*Group V*: Healthy persons lived in a disease-endemic area according to the central patient registry of our institution, Southern Province (endemic healthy controls/EHC, *n* = 50).

The absence of leishmaniasis in control samples was confirmed based on absent clinical picture and/or confirmed alternative diagnosis and response to appropriate treatment in NCL, EHC, and NEHC or based on negative clinical picture and/or negative LM/IVC/PCR of skin or bone marrow samples in CL, NCL, and NVL.

### 2.2. Sample Collection and Preparation of Serum Samples

Venous blood (3 cc) was collected by trained medical or paramedical personnel after obtaining an informed written consent from each patient and control persons. Blood samples were incubated at room temperature for 30 minutes to 1 hour to allow the clotting of blood. Serum was separated by centrifugation at 2500 rpm for 10 minutes and aliquoted and stored at -20°C for later use.

### 2.3. Crude Antigen Extraction

A locally acquired, confirmed positive CL sample was selected from the sample cohort. If positive patients were with a history (within two years prior to diagnosis) of overseas travel, they were excluded from the study. M199 supplemented with 10% HI-FBS and 1% PenStrep (M199-complete media) was used for *in vitro* culturing procedures. Once parasite count reached to late log phase (1 × 10^7^ cells/ml), cultures were used for harvesting parasites.

Total crude extracts were prepared from harvested promastigotes of *L. donovani.* Pellet was washed three times in cold 1× phosphate-buffered saline (PBS), pH 7.4, and resuspended at a concentration of 1.0 g of cell pellet in 2 ml of 1XPBS, pH 7.4. Suspension was freeze-thawed for three times (freezed in liquid nitrogen and thawed at room temperature). Crude Ag was quantified using a modified Lowry assay [25]. It was aliquoted and stored at -20°C.

### 2.4. ELISA

ELISA was carried out using a previously optimized protocol [20, 26, 27]. A ninety-six- (96-) well ELISA plate (Sterilin or Greiner) was coated with 100 *μ*l (containing at least 1 *μ*g protein) of crude protein extract and incubated overnight at 4°C. The plate was washed three times with 1× PBS supplemented with 0.1% tween-20 (PBST) and treated with 200 *μ*l of 2% 1× PBS-FBS (2 ml of FBS in 100 ml of 1XPBS) for blocking reaction wells and incubated at room temperature for 6 to 8 hours. Patient sera at 1 : 1000 dilution was added and left overnight at 4°C. Following overnight incubation, the plate was washed with PBST for three times and incubated with secondary antibody (Goat anti-human IgG- (total-) HRP) in 1 : 64000 dilution (100 *μ*l/well) at 37°C for 30 minutes. The plate was washed again with PBST for six times with 5 minutes intervals with gentle shaking for the last five washings. Subsequently, the plate was incubated with 100 *μ*l of TMB substrate solution at room temperature for 30 minutes, and the reaction was stopped by adding 100 *μ*l of 1 N H_2_SO_4_. The absorbance was read at 450 nm using an ELISA reader (Epoch 2 microplate spectrophotometer from BioTek instruments).

### 2.5. Quality Control and Analysis of Data

Each sample was analyzed in duplicates, and the mean value of absorbance was considered as the final value. Only absorbance values closer to the second decimal point in duplicates were considered in calculating mean. Each ELISA plate was run with an air blank, five or more healthy controls and controls with and without conjugate. Normalization of day-to-day variations of the assay and test reproducibility was assessed according to an acceptance and rejection criteria defined using mean absorbance values of healthy controls (*M*_healthy_). According to that criteria, the mean absorbance value of healthy controls in each ELISA run should be within the range of *M*_healthy_ ± 2(standard deviation, SD)_healthy_. If more than four healthy controls fell outside the range, the test was rejected and repeated.

### 2.6. Validation of ELISA

Validation of ELISA was carried out according to approved guidelines described on fundamental validation parameters for immunoassays which were presented in U.S. Pharmacopeia Chapter 1225, Validation on Compendial Methods, 2009 and ICH Q2 (R1) on Validation of Analytical Procedures: Text and Methodology, 2005 [28].

#### 2.6.1. Sensitivity, Specificity, Negative Predictive Value, and Positive Predictive Values of ELISA

The cut-off value for the assay was determined using a receiver operating characteristic curve (ROC curve). The area under curve (AUC) was determined using the ROC curve. AUC values closer to one are considered as tests with high diagnostic accuracy which reliably distinguishes positive and negative samples. Sensitivity, specificity, negative predictive value (NPV), and positive predictive value (PPV) of assay were calculated using the ROC curve and 2 × 2 table analysis. All cases and control groups were included in the analysis of the ROC curve including CL (*n* = 200), NCL (*n* = 50), NVL (*n* = 50), NEHC (*n* = 50), and EHC (*n* = 50). In ROC curve analysis using SPSS (version 25.0) statistical software, ELISA absorbance values of each sample were used as test variable, and the positivity of samples for CL were used as state variable.

#### 2.6.2. Linearity

The reference standard used for quality controlling of assay was obtained from a patient with Indian VL. The serum was positive for the rK39 strip test. Linearity of the assay was determined using a standard curve constructed with six analyses of five different concentrations (concentration spanned from about 80%-120% of expected concentration range, i.e., 1 : 16000, 1 : 32000, 1 : 64000, 1 : 128000, and 1 : 512000) of reference standard

#### 2.6.3. Reproducibility/Repeatability

More than six determinations of three different matrices at three different concentrations were performed, and relative standard deviation was calculated to determine reproducibility/repeatability. Accordingly, ELISA values obtained for healthy controls within 20 different days were analyzed. Also, relative standard deviation (SD) and coefficient of variation (CV, <10 of CV was considered as highly accurate with high reproducibility/repeatability) of ELISA values for high and intermediate positive sera within 10 days were calculated.

#### 2.6.4. Accuracy

The accuracy of the test was also determined in relation to gold standard, i.e., LM using standard calculation methods (Accuracy = true positives and true negatives/total number of samples). The mean reactivity of CL sera was further compared with other control groups, and statistical significance was calculated using SPSS version 25.0.

#### 2.6.5. Limit of Blank (LOB), Limit of Detection (LOD), or Limit of Quantitation (LOQ)

The smallest concentration of a measurand that can be reliably measured by test was determined using standard ELISA parameters, i.e., LOB, LOD calculated using standard equations, LOB = *M*_Blank_ + 1.645 (SD_Blank_), LOD = LOB + 1.645(SD_Low concentration of analyte_) [29]. Fifty ELISA done on different dates were used for the determination of LOD.

#### 2.6.6. Range

According to manufacturer recommendations of ELISA reader used, the accurate range was typical value ±1% (0–2.0 Abs) at 405 nm.

#### 2.6.7. Stability of Samples

Samples were stored in aliquots for avoiding repeating freeze-thawing cycles. Also, the same room conditions, temperature, and light/dark conditions were used for each run to increase the accuracy of the test. Samples, stock solutions, and other reagents were stable and used for more than two years without any deviation of ELISA readings by aliquoting and storing under recommended conditions.

#### 2.6.8. Comparison of ELISA with Parasitological Diagnostic Methods

To further validate ELISA, assay results were compared with classical parasitological diagnostic methods used for CL, i.e., LM and IVC. Also, cost analysis per patient was carried out according to approved guidelines [30]. Expenses for laboratory consumables, chemicals, and reagents were estimated according to their current cost in USD. Expenses for laboratory personnel and equipment were not considered for analysis.

#### 2.6.9. Clinicoepidemiological Correlations of ELISA

Serological response was further compared with the epidemiological data of patients (i.e., age and sex) and clinical features of lesions (i.e., lesion duration, number, type, size, and site) using SPSS version 25.0.

### 2.7. Ethics Statement

Samples were collected upon written informed consent given by patients and healthy controls. Ethics approval for the study was obtained from the Ethics Review Committee, Faculty of Medicine, University of Colombo.

## 3. Results

### 3.1. Validation of ELISA

#### 3.1.1. Sensitivity, Specificity, Negative Predictive Value, and Positive Predictive Values of ELISA

**Figure 1:**
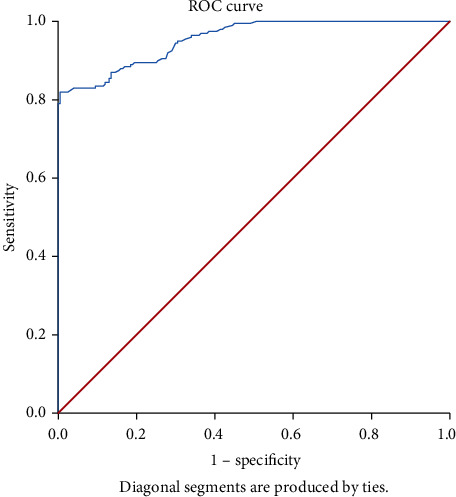
ROC curve for ELISA. ELISA absorbance that maximized the total of sensitivity and specificity was selected as the best cut-off value.

*(1) ROC Curve Analysis*. According to results obtained for the ROC curve (Figure 1), AUC was 0.955 with a standard error of 0.009 and a 95% confidence interval from 0.938 to 0.972.

According to results obtained for ROC analysis, the best cut-off value was 0.189 of absorbance. At this cut-off, sensitivity is 82.0% and specificity is 99.5% (Table 1).

#### 3.1.2. Analysis of Diagnostic 2 × 2 Table

**Figure 2:**
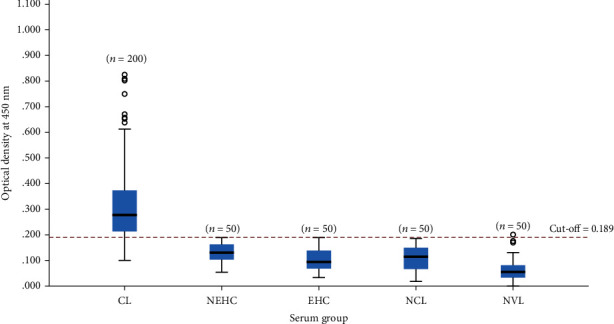
Optical density distribution of ELISA. Variation of ELISA values of CL and control groups including NEHC, EHC, NCL, and NVL is shown at 0.189 cut-off level.

At 0.189 cut-off value obtained from the ROC curve, of 200 CL samples studied, in-house ELISA identified 164 seropositive cases (*n* = 164/200, 82.0%) at a OD value greater than 0.189. None of the sera from NCL, NEHC, and EHC was seropositive for ELISA except for 1 NVL sample (Figure 2). Therefore, according to the 2 × 2 table shown in Table 2, PPV and NPV were 99.4% and 84.7%, respectively.

#### 3.1.3. Linearity

**Figure 3:**
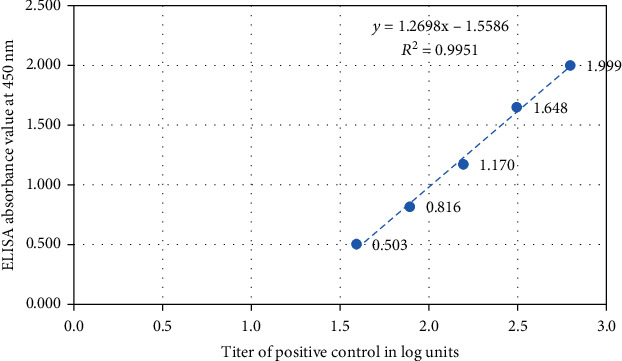
Standard curve for a dilution series of reference standard. Five known concentrations of reference standard were used.

According to the standard curve shown in Figure 3, two variables of assay, Ab concentration, and ELISA value showed a linear relationship where the squared correlation coefficient, *R*^2^, was 0.9951.

#### 3.1.4. Reproducibility/Repeatability

**Figure 4:**
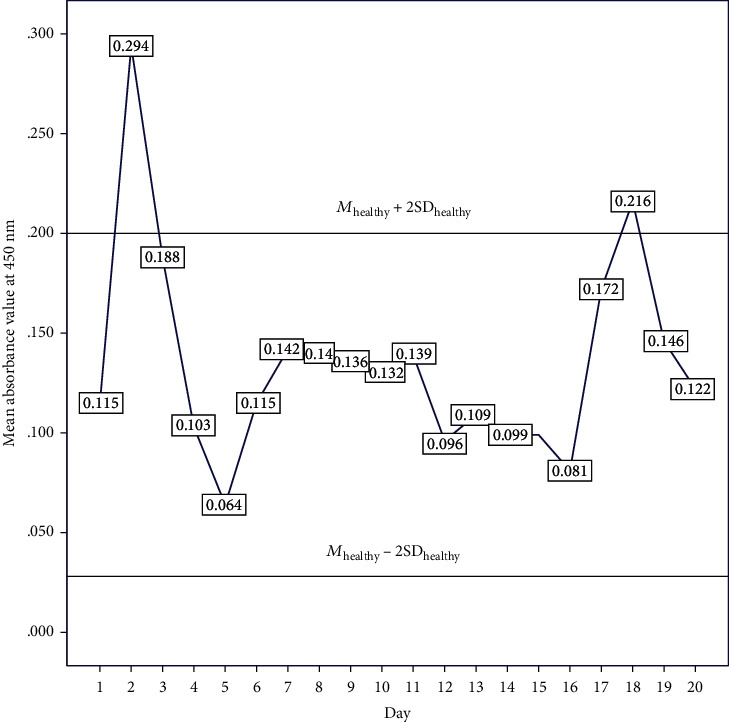
Variation of mean value of ELISA absorbance at 450 nm obtained for healthy controls. *M*_healthy_ + 2SD_healthy_ and *M*_healthy_ − 2SD_healthy_ represent upper and lower limits, respectively.

*M*_healthy_ was calculated as *M* = 0.114 and SD_healthy_ was 0.043. Therefore, *M*_healthy_ + 2SD_healthy_ = 0.200 and *M*_healthy_ − 2SD_healthy_ = 0.028. There were *n* = 2/20 samples that had ELISA values beyond the upper limit and thus reproducibility of the test was 90% (Figure 4).

CV of ELISA values for high and intermediate positive sera within 10 days period was about 2.6% and 3.5%, respectively (Table 3).

#### 3.1.5. Accuracy

The accuracy of the assay was determined as 90.8%.

Mean reactivity for sera from CL patients was statistically different to those from healthy individuals (NEHC and EHC) and patients with other diseases (NCL and NVL) (*p* ≤ 0.001) (Table 4).

#### 3.1.6. Limit of Blank (LOB), Limit of Detection (LOD), or Limit of Quantitation (LOQ)

*M*_Blank_ was determined as 0.048 and LOB was calculated as 0.060. Therefore, LOD or LOQ of the assay was 0.131.

#### 3.1.7. Comparison of ELISA with Parasitological Diagnostic Methods

There were *n* = 102 LM positive cases. Among them, *n* = 86/102 (84.3%) were ELISA positive. Among LM negative group of *n* = 16 cases, *n* = 12/16 (75.0%) cases gave positivity for ELISA (Table 5(a)). Also among *n* = 70 of total IVC positive cases, *n* = 58/70 (82.9%) were ELISA positive. Among the IVC negative group of *n* = 48 cases, there were *n* = 40/48 (83.3%) ELISA positive cases (Table 5(b)). According to the cost analysis, cost per patient was 3.0 USD for both ELISA and LM and 6.0 USD for IVC.

### 3.2. Clinicoepidemiological Characteristics of Seropositive Group of CL Patients

Patients presented during the period from 2002 to 2017. More than 75.0% of CL infections in each gender demonstrated seropositivity. The majority of infections occurring in all age groups also demonstrated a seropositivity of >75%. Lesions of short (<3 months), medium (4 to 6 months), and long durations (>6 months) were also associated with high seropositivity (72-92.0%), with lesions of very short duration (<3 months) also showing >70% seropositivity (Table 6). Over 70% of lesions of different sizes also demonstrated a seropositivity while ELISA was positive in the majority of very early lesions also (<1 cm diameter) (*n* = 34/49, 69.4%). Similarly, ulcerative and nonulcerative lesions and single and multiple lesions were also associated with >70% seropositivity. Lesions occurring on the head and neck area demonstrated a slightly lower seropositivity rate as compared to lesions on limbs and other sites (66.7 vs. 86.2 and 87.2).

Both genders and a wide age range in CL cases responded satisfactorily with a high serological response. At the selected cut-off level, there were no age or gender-dependent significant reduction in seroprevalence (*p* > 0.05).

## 4. Discussion

This is the first time study reporting a high seroprevalence (82.0% at 99.5% specificity) in CL caused *L. donovani*. A high (>70%) serological response was seen in the majority of CL infections in all age groups, both genders, and in all studied lesion types in this study. But it is usually the VL infection that gives high seroprevalence [31–33]. Compared to seroprevalence rates reported for CL in other endemic settings in the world, the new assay described here reported a high value [34–36]. As instances, ELISA developed by Zeyrek et al. showed 78.0% and 95.3% of sensitivity and specificity, respectively [34]. In Szargiki et al., sensitivity and specificity were about 71.7% and 84.6% when using *L. amazonensis* as Ag and 95.0% and 92.3% when using *L. braziliensis* as Ag [35]. In Sarkari et al., it was 83.6% and 62.7% [36]. Also, studies done on serology for *L. infantum* or *L. donovani* causing CL were limited and they showed less than 50% seroprevalence with rK39 dipstick assay [37, 38]. About 0.955 of AUC value, 90.8% test accuracy, and statistically significant absorbance values (Table 4) obtained for each category of patients (CL, NCL, and NVL) and control group (NEHC and EHC) in this study further demonstrated the high accuracy of ELISA developed in the present study.

This high level of seroprevalence could be due to a still unconfirmed potential for visceralization or a transient serological response associated with localized CL infections.

*L. donovani*, *L. infantum*, and *L. chagasi* which are the members of *L. donovani* complex usually results in VL, while cutaneous lesions attributed to these have also been reported [13–15, 38–40]. Though CL is generally not considered to evoke a humoral immune response, seropositivity obtained in this study indicates different possibilities of local CL such as its potential to visceralize [41–45], simultaneous antibody reaction without visceralization [35, 36, 46–48], a serological response found together with CL, a post-kala-azar dermal leishmaniasis (PKDL) like illness [49–53], and asymptomatic coinfection with VL strains [54–57].

Some studies have rejected these possibilities of CL having lower seroprevalence in CL. As instances, in Svobodova et al., CL patients caused by *L. infantum* were negative in rK39 test confirmed nonvisceral form of leishmaniasis [38], in Molinet et al., lack of cross-reaction in 100% of samples for rK39 test that were analyzed in this study highlights high specificity for patients with LCL (localized CL infections) in areas that are endemic for *L. (V.) braziliensis* [58]. Also in Sharma et al., antibody response to rK39 was largely VL-oriented (*L. donovani-infantum* complex) [37]. There was no response in infection with *L. tropica* (CL) (Sharma and Singh 2009). Positive rapid rK39 immunochromatographic dipstick testing in two VL (100%) and four LCL (31.8%) patients suggested the presence of *L. donovani-infantum* infection in this endemic focus.

Analysis of clinicoepidemiological characteristics of patients further highlighted some associations of lesion site, type, duration, and gender of patients with seropositivity (Table 6). These findings were in agreement with other studies and further explained phenotypic-based variations seen in humoral response in CL patients [34, 36, 47, 59, 60]. Zeyrek et al. observed a positive correlation between seropositivity and clinical properties (lesion size, lesion location, and lesion type) [34]. A positive correlation between seroprevalence and the number of lesions in a patient has been previously reported [34, 47].

Low seropositivity in patients having lesions in the head and neck area may be due to a higher amount of lymph nodes of human body area are located in the head and neck area, leading to higher cell-mediated immunity compared to humoral immunity [61]. Also, aggravation of inflammatory reactions observed with lesion ulceration usually leads to produce increased levels of regulatory cytokines (i.e., TGF-*β* and IL-10) [62]. It may subsequently enhance B-cell survival, proliferation, and antibody production within the body. High expression of IgG in later stages compared to early lesions and higher levels of mean IgG levels in males compared to females may cause for high seropositivity observed in late lesions and in males, respectively [63, 64].

Diagnosis of CL and other clinical forms based on clinical presentations is challenging in tropical settings due to the presence of many other conditions with similar clinical profiles. With a high level of specificity, assay can be useful in differentiating leishmanial infections from other nonleishmanial conditions. PPV and NPV of ELISA were 99.4% and 84.7%, respectively. High PPV allows the assay to remain useful even when the prevalence of leishmaniasis is low or decreasing. A good NPV allows an accurate diagnosis at a high specificity.

In addition, this ELISA assay seems to be suitable in detecting CL infections in all studied age and gender categories with *L. donovani*-induced CL infections in Sri Lanka. ELISA was also useful in detecting all clinical stages of a lesion. A clear majority of both single and multiple lesions, lesions of different type, duration, and size when analyzed separately remained highly (>70%) seropositive. It is often difficult to sample small and early papular types of lesion in order to carry out parasitological investigations. In addition, patients with these types of lesions are less likely to seek early medical care due to their nondisturbing nature. This highlights the importance of field-level screening and detection of early lesions. ELISA performed on a serum sample can be more convenient as compared to parasitological investigations that require sampling a skin lesion by a trained person in the field setting.

ELISA assay was able to detect 75.0% of LM negative cases. Also, in ELISA negative cases, only 80.0% was detected by LM or 60.0% by IVC. Parasitological investigations can become negative in chronic, treated, atypical, or partially treated infections. However, in order to assess treatment response and cure, it is important to establish a laboratory-based diagnosis in all possible cases. Positivity obtained in ELISA was comparable to LM but higher than IVC. Since IVC needs invasive sample collection procedures, experts to handle, and it is highly possible with contaminations, ELISA can be used as an additional diagnostic method for local CL. Presumably, ELISA will replace IVC with the added advantage of low cost and noninvasiveness. Also, ELISA will be useful where infection cannot be detected by eliciting the presence of parasites or parts of them. Therefore, in-house ELISA could be used as a useful second line option in the detection of all LM-negative cases before expensive and complex IVC or molecular biological procedures are performed. Furthermore, assay cost is also comparable to that of LM, which is the first line investigation used in routine case detection in leishmaniasis. In addition, ELISA could further be performed without having to sample an infection site.

Asymptomatic infections comprise an important component that contributes to the silent onward propagation of disease in leishmanial endemic settings. Increasing efforts are made to study this clinical entity in affected countries, and most studies employ serological tools due to lack of obvious bodily sites of infection that hinder the researcher from collecting infected tissue for parasitological assessment. The usefulness of newly developed assay could be explored in the detection of preclinical infections in Sri Lanka and similar settings. Furthermore, this assay could be useful in the detection of other recently emerged visceral and mucosal leishmaniasis in Sri Lanka. Further understanding on immune dominant antigen profile, further associations with clinical and parasitological variations, and usefulness as an early detection or outcome prediction marker will be useful.

With several evidences associated with CL and seropositivity in the world as mentioned above, it is still unknown whether the seropositivity observed in local CL due to visceralization potential of the local parasite or immunogenic nature of the parasite. The developed ELISA will be useful as a second line investigation for increasing the successive case detection rate of local CL in the near future.

*Applications were submitted for patenting at National Intellectual Property Office of Sri Lanka (National patent LK/P/1/19697).*

## Figures and Tables

**Table 1:** Sensitivity and (1-specificity) for ELISA at different absorbance values. Italics show the best cut-off value.

Positive if greater than or equal to (ELISA absorbance values)	Sensitivity	1-specificity	Specificity	Sensitivity +specificity
0.181	0.830	0.065	0.935	1.765
0.182	0.830	0.050	0.950	1.780
0.183	0.830	0.045	0.955	1.785
0.184	0.830	0.040	0.960	1.790
0.185	0.820	0.025	0.975	1.795
0.187	0.820	0.015	0.985	1.805
*0.189*	*0.820*	*0.005*	*0.995*	*1.815*
0.191	0.815	0.005	0.995	1.810
0.192	0.805	0.005	0.995	1.800
0.196	0.800	0.005	0.995	1.795
0.199	0.795	0.005	0.995	1.790
0.200	0.790	0.005	0.995	1.785
0.201	0.790	0.000	1.000	1.790
0.202	0.785	0.000	1.000	1.785

**Table 2:** Diagnostic 2 × 2 table for in-house ELISA.

		Disease confirmatory status			
		Positive for CL	Negative for CL	Total count	
In-house ELISA	Seropositive	164	1	165	PPV = 164/165 = 99.4%
	Seronegative	36	199	235	NPV = 199/235 = 84.7%
	Total count	200	200	400	
		SE = 164/200 = 82.0%	SP = 199/200 = 99.5%		

**Table 3:** Repeatability assay using high and low positive serum samples for 10 days.

Replicates	ELISA value for high positive serum	ELISA value for low positive serum
1	0.449	0.222
2	0.430	0.226
3	0.447	0.219
4	0.420	0.219
5	0.419	0.223
6	0.427	0.2055
7	0.441	0.207
8	0.419	0.22
9	0.418	0.2175
10	0.428	0.232
M	0.430	0.219
SD	0.011	0.008
CV	2.6	3.5

**Table 4:** Comparison of mean ELISA values obtained for sera of CL patients, healthy individuals (NEHC and EHC), patients with other skin diseases (NCL), and patients with other systemic diseases (NVL).

Serum samples	ELISA absorbance, mean ± SD	Confidence interval of 95%		*p* value (CL versus other sera group)
		Lower limit	Upper limit	
CL	0.305 ± 0.139	0.286	0.324	
NEHC	0.129 ± 0.039	0.118	0.140	≤0.001
EHC	0.099 ± 0.042	0.087	0.111	≤0.001
NCL	0.111 ± 0.050	0.097	0.125	≤0.001
NVL	0.063 ± 0.044	0.051	0.075	≤0.001

**Table:** (a) Comparison of results obtained for ELISA with LM

		LM positive	LM negative	Total count
ELISA	Seropositive	86	12	98
	Seronegative	16	4	20
	Total count	102	16	118

**Table:** (b) Comparison of results obtained for ELISA with IVC

		Culture positive	Culture negative	Total count
ELISA	Seropositive	58	40	98
	Seronegative	12	8	20
	Total count	70	48	118

**Table 6:** Clinicoepidemiological correlations with seropositivity in the study populations.

	Seroprevalence measured by ELISA^∗^			
Clinicoepidemiological data	Seropositive count (*n*%)	Seronegative count (*n*%)	Total count (*n*)	*p* value
Age (years)				
≤25	38/49 (77.6)	11/49 (22.4)	49	0.497
26 to 50	96/118 (81.4)	22/118 (18.6)	118	
>50	29/33 (87.9)	4/33 (12.1)	33	
Sex				
Male	133/160 (83.1)	27/160 (16.9)	160	0.257
Female	30/40 (75.0)	10/40 (25.0)	40	
Lesion size^∗∗^				
Up to 1 cm	34/49 (69.4)	15/49 (30.6)	49	0.044
2-3 cm	89/103 (86.4)	14/103 (13.6)	103	
>3 cm	34/42 (81.0)	8/42 (19.0)	42	
Number of lesions				
One lesion	133/161 (82.6)	28/161 (17.4)	161	0.490
>one lesion	30/39 (76.9)	9/39 (23.1)	39	
Duration of lesions^∗∗^				
Up to 3 months	46/64 (71.9)	18/64 (28.1)	64	0.017
4 to 6 months	56/61 (91.8)	5/61 (8.2)	61	
>6 months	44/55 (80.0)	11/55 (20.0)	55	
Type of lesions^∗∗^				
Ulcerative^#^	95/108 (88.0)	13/108 (12.0)	108	0.008
Nonulcerative^#^	65/89 (73.0)	24/89 (27.0)	89	
Site of lesion^∗∗^				
Head and neck area	36/54 (66.7)	18/54 (33.3)	54	0.007
Arms	81/94 (86.2)	13/94 (13.8)	94	
Other	41/47 (87.2)	6/47 (12.8)	47	

^∗^Seroprevalence was measured by ELISA at 0.189 cut-off level. ^∗∗^Missing data were excluded. ^#^Working definitions: lesions on the skin accompanied by the disintegration of tissue or not were considered as ulcerative (viz., ulcerating nodules, ulcerating plaques, and complete ulcers) and nonulcerative (viz., papules, nodules, and plaques) lesions, respectively.

## Data Availability

Data supporting the conclusions of this article are included within the article. Other data has not been made available as it was not part of the ethics application and due to patient confidentiality.
